# Gain of Chromosomal Region *3q26* as a Prognostic Biomarker for High-Grade Cervical Intraepithelial Neoplasia: Literature Overview and Pilot Study

**DOI:** 10.1007/s12253-018-0480-y

**Published:** 2018-10-25

**Authors:** Margot M Koeneman, Irene T Ovestad, Emiel A. M. Janssen, Monique Ummelen, Roy F. P. M. Kruitwagen, Anton H. Hopman, Arnold J. Kruse

**Affiliations:** 1 Department of Obstetrics and Gynecology, Maastricht University Medical Centre, Maastricht, the Netherlands; 2 GROW - School for Oncology and Developmental Biology, Maastricht University, Maastricht, the Netherlands; 3 Department of Pathology, Stavanger University Hospital, Stavanger, Norway; 4 Department of Mathematics and Natural Science, University of Stavanger, Stavanger, Norway; 5 Department of Molecular Cell Biology, Maastricht University, Maastricht, The Netherlands; 6 Department of Obstetrics and Gynecology, Isala Clinics, Zwolle, The Netherlands

**Keywords:** *hTERC*, *3q26*, Cervical intraepithelal neoplasia, Prognosis, Biomarker

## Abstract

Approximately 20–40% of high-grade Cervical Intraepithelial Neoplasia (CIN) regresses spontaneously, but the natural prognosis of an individual lesion is unpredictable. Gain of the chromosomal 3q region, which contains the human telomerase RNA gene on *3q26*, is found in CIN lesions and cervical carcinoma and shows correlation with disease grade. The aim of this study is to assess whether *3q26* gain as a single genetic marker can predict the natural prognosis of high-grade CIN, by performing a review of the literature and pilot study. A literature review was conducted. Additionally, we performed a pilot study in 19 patients with histologically confirmed high-grade CIN lesions who were followed for a mean of 115 days, after which loop excision was performed. Fluorescent in situ hybridization analysis was performed on the initial diagnostic biopsies to determine gain of *3q26*. Eight studies were included in the literature overview, with a total of 407 patients. Of these, only 22 patients had high-grade lesions. All studies found an association between *3q26* gain and disease prognosis. Positive predictive values (PPV) ranged from 50 to 93%, negative predictive values (NPV) ranged from 75 to 100%. Only five out of 155 patients (3.2%) without *3q26* gain showed disease persistence or progression. In our pilot study on *3q26* gain in high-grade CIN, the PPV of *3q26* gain for disease persistence was 67%, the NPV 100%. All four patients without *3q26* gain showed disease regression. In conclusion, the absence of *3q26* gain in diagnostic biopsies may be applied to identify high-grade CIN lesions with a high probability of disease regression.

## Introduction

High-grade Cervical Intraepithelial Neoplasia (CIN) is caused by Human Papillomavirus (HPV)-infection and is considered to be the precursor of cervical carcinoma [1]. Approximately 30% of high-grade lesions progresses to cervical cancer on the long term, whereas spontaneous regression occurs in approximately 20–40% [2–6]. Conventional histopathological assessment is unable to differentiate between high-grade lesions that will progress to cervical cancer and those that will regress spontaneously. Consequently, most high-grade lesions are currently treated, leading to significant overtreatment with associated side-effects [7]. Ideally, the natural prognosis of individual CIN lesions would be predictable, in order to select patients in whom spontaneous regression is expected for a wait-and-see policy.

It has been established that the development of CIN and concurrent progression to cervical cancer is influenced by a complex interaction between HPV, the host immune system and functional cellular mechanisms [8, 9]. Cervical oncogenesis is characterized by several genetic effects, among which are genomic instability, chromosomal aberrations and integration of viral DNA into the host genome. Markers of these processes have been identified as potential diagnostic or prognostic biomarkers in the diagnosis and prognosis of CIN [10, 11]. Among these is chromosomal region 3q gain, which is frequently found in cervical carcinomas and its precursor lesions [12]. The association between 3q gain and cervical oncogenesis may be caused by amplification of the human telomerase RNA gene (*hTERC*), which is localized on the *3q26* locus. The *hTERC* gene encodes for the RNA unit of telomerase, which maintains the length of telomeres through cellular divisions. Overexpression of *hTERC* leads to the avoidance of abnormal cells with critically short telomeres to undergo apoptosis, which is a contributing factor in oncogenesis. Gain of *3q26/hTERC* or copy number variations has been shown to correlate with disease grade in cervical lesions and could function as a diagnostic tool in cervical pathology [13–16]. Several studies have addressed the prognostic properties of *3q26/hTERC* gain in the natural prognosis of CIN, but most studies focussed on low-grade lesions and/or evaluated 3q gain in cytological specimen. Evidence on 3q gain in histologically confirmed high-grade CIN is very scarce. The goal of this study is to provide an overview of the literature on the prognostic properties of *3q26/hTERC* gain in the natural prognosis of CIN and to investigate the predictive properties of *3q26* gain specifically in high-grade CIN.

## Materials and Methods

The study was performed according to the PROBE criteria for biomarker research, where possible and applicable.

### Patient Population

For the pilot study, the patient population was extracted from a prospective population based cohort study, conducted at the Stavanger University Hospital, Norway [5]. The women in this cohort were diagnosed with a CIN2 or CIN 3 lesion in a diagnostic biopsy. All biopsies were stained for Hematoxylin Eosin, p16 and Ki-67 and disease grade was based on the most severely dysplastic area with the most intensive Ki-67 and p16 staining. Staining was assessed for disease grade by consensus scoring of three observers, followed by independent quality control of a fourth observer. All used the same microscope (40 × objective 0.52 mm, numerical aperture 0.65). All women underwent a Loop Electrosurgical Excision Procedure (LEEP) after a median of 113 days follow-up (range 84–171 days). The natural history of the baseline cervical lesion during the follow-up period was evaluated in the LEEP specimen. Regression was defined as CIN1 or less in the LEEP specimen. Further details on histological evaluation, HPV genotyping and lesions size measurements can be found in the original article [5]. Out of this cohort, representative and sufficient baseline biopsy material for *3q26* analysis was available for 19 patients. These patients were included in the pilot study.

### FISH Procedure

FISH analysis was performed on the baseline biopsies. The 3q specific FISH was performed on 4 μm thick FFPE tissue sections fixed onto Superfrost Plus Microscope Slides (Thermo Fisher Scientific). The tissue sections were first heated for 15 min at 80 °C, then dewaxed, hydrated and microwaved for 10 min at 100 °C in a 10 mM Na-Citrate pH buffer and incubated at room temperature for 20 min to cool down. Subsequently, the sections were washed in demineralized water, rinsed in 0.01 M HCl and digested with 2.5 mg of pepsin in 0.01 N HCl and post-fixed in 1% formaldehyde in PBS for 5 min at room temperature. Subsequently, the 3 centromere probe (pα3.5) and *3q26* probe (*3q26*.1: BAC23 RP11-264D7, Map position *3q26*.1–26.3 close to the TERC locus), were labeled with Digoxigenin (3c) and Biotin (3q) in a nick translation labelling (Jena Bioscience GmBH, Jena, Germany). The probes were hybridized at a concentration of 2 ηg/μl (3c), 5 ηg/μl (3q); 10 x excess COT, and 75x excess of carrier DNA (salmon sperm DNA) in 50% formamide; 2x SSC; 10% dextran sulphate. The probe was applied under a coverslip, simultaneously denatured for 10 min at 80 °C and hybridized overnight at 37 °C. After hybridization, the preparations were washed for 5 min at 61 °C in a solution, containing 2 × SSC, 0.05% tween-20 (Janssen Chimica, Beerse, Belgium) and 0.1 × SSC (the washing was carried out twice). The hybridized FISH probe was detected with a triple layer detection method, consisting of 1. FITC-conjugated avidin (Av-FITC, 1:100 dilution, Vector Laboratories) / Monoclonal anti-Digoxigenin (MαDig, 1:100 dilution, Sigma, USA, St Louis MO); 2. Botinylated Goat anti-Avidin (Bio-GαA, 1:100 dilution, Vector Laboratories USA) / Rabbit anti Mouse-TRITC (1:100 dilution, Dako, Glostrup, Denmark) and 3. Av-FITC / Swine anti Rabbit-TRITC (1:100 dilutionDako). Finally, the slides were washed in PBS containing 0.05% Tween-20, dehydrated in an ascending ethanol series and mounted in Vectashield (Vector Laboratories), containing DAPI (Sigma: 0.5 μg/μl). Images were recorded with the Metasystems Image Pro System (black and white CCD camera; Sandhausen, Germany), mounted on top of a Leica DM-RE fluorescence microscope [15].

### FISH Evaluation

The FISH signals were interpreted by two analysts (MU, AH), who were blinded to the outcome data. Dysplastic areas were identified based on p16 staining and were scanned for FITC and TRITC signal copy numbers. The copy number was estimated as previously described to detect disomy, tetrasomy up to nonasomy by means of the determination of the maximum copy number and heterogeneity in formalin fixed and paraffin embedded tissue sections. The validity of this strategy was independently proven by means of a statistical analysis of spot counting in tissue sections [17, 18]. Lesions were classified are tetrasomic (copy number 4) in case a major fraction exhibited 4 copies, as aneusomic (copy number 3,4) in case a major fraction exhibited 3 copies and in case of minor fractions (copy number 2–4). Gain for the targets was noted when dysplastic areas were recognized with more than two copies for 3c or 3q. Subsequently, normal morphologic areas were analysed and consistently showed a disomy for 3c and 3q.

### Outcome Measures and Criteria for Biomarker Performance

The outcome measure was defined as the correlation between *3q26* gain and disease persistence of high-grade CIN. No previous biomarker performance values are available for HLA types. The required test performance values include a high sensitivity and negative predictive value: lesions that will not regress spontaneously must be identified, as treatment is necessary in these women. The actual values depend on the follow-up term of observational management. Lower values can be accepted when strict histological follow-up is implemented to identify persisting lesions at an early stage.

### Outcome Measure and Statistical Analysis

Quantitative variables were described as mean and ranges. Qualitative variables were described as frequency and percentage. Sensitivity, specificity, positive and negative predictive values were calculated from a 2 × 2 table. Sample size calculation was not feasible, due to the lack of comparable biomarker performance values and limitation of the study population by the availability of material.

## Literature Overview

Eight studies were identified that evaluated the predictive properties of *3q26/hTERC* gain in cervical squamous lesions [19–26]. All studies assessed patients with cervical squamous intraepithelial lesions who were followed for a certain period of time, without immediate treatment, in order to evaluate the natural prognosis of the lesions. The main study features are displayed in Table 1. Only two studies included patients with high-grade lesions. Heselmeyer-Haddad included patients with German PAP 3D cytology, which resembles CIN 1 or 2, of which the latter is interpreted as a high-grade lesion [19]. Ravaioli et al. included five patients with high-grade CIN [26].

**Table 1:** Main features of studies included in the review

Study	Baseline pathology	No. of cases	Specimen	Follow-up term	Follow-up measure	HPV tested?	Distinction polyploidy/ gain	No. of evaluated cells	Cutoff	Cases with gain	Disease outcome
Heselmeyer-Haddad 2005 [19]	German PAP3D cytology (resembling CIN^a^ 1/2)	22	Liquid-based cytology	2 m – 2y	Cytology	No	Yes	Different for each case: based on cell density and focused on aberrant cells	> 2 signals in >20% of cells(pragmatic)	Gain: 7/22 (32%) Gain/tetraploidy: 15/22 (68%)	Remission: 10/22 (45%) Progression: 12/22 (55%)
Alameda 2009 [20]	LSIL^b^ cytology	30*	Liquid-based cytology	6, 12 and 24 m	Cytology followed by colposcopy with biopsy on indication	Yes	No	Min 400	> 2 signals in >1.6% of cells (based on mean gain in control population)	19/30 (63%)	Remission: 6 m: 13/30 (43%) 12 m: 18/26 (69%) 24: 13/21 (62%) Persistence/progression: 6 m: 17/30 (57%) 12 m: 8/26 (31%) 24 m: 8/21 (38%)
Jalali 2010 [21]	LSIL cytology	47	Liquid based cytology	< 12 m – 85 m	Cytology (*n* = 16) or biopsy (*n* = 31)	No	Yes, tetraploidy ruled out by defining gain as >4 signals	15–20 HPF	>4 signals in ≥2 cells, pragmatic	17/47 (36%)	Non-progression: 36/47 (77%) Progression: 11/47 (23%)
Lan 2012 [22]	CIN 1–2, histological diagnosis	54	Liquid-based cytology	24 m	Colposcopy with biopsy	Yes	No	Min 100	> 2 signals in >5.48% of cells (based on mean gain +3SD of control population)	27/54 (50%)	Regression: 20/54 (37%) Persistence: 21/54 (39%) Progression: 13/54 (24%)
Rodolakis 2012 [23]	CIN1 or koilocytosis, histological diagnosis	40 (31 LSIL and 9 ASCUS^c^)	Liquid based cytology	11-22 m	Cytology and colposcopy with biopsy	No	Yes, tetraploidy ruled out by defining gain as >4 signals	800 cells with the highest number of signals	>4 signals in ≥2 cells OR > 4 signals in ≥1 cell and 10 cells with 4 3q signals and 2 centromere 7 signals (4–2), pragmatic	8/40 (20%)	Non-progression: 37/40 (93%) Progression: 3/40 (8%)
Obermann 2013 [24]	LSIL cytology	132	Liquid-based cytology	Min 6 m	Cytology followed by colposcopy with biopsy on indication	Yes	No	Up to 50 cells	> 2 signals in ≥10% of cells, pragmatic	LSIL: 31% (absolute numbers not reported)	Regression: 67/132 (51%) Persistence: 55/132 (42%) Progression: 10/132 (8%)
Li 2014 [25]	CIN 1, histological diagnosis	74	Liquid based cytology	24 m	12 m: HPV test 24 m: colposcopy with biopsy and HPV test	Yes	No	100 cells	>2 signals in >6% cells (based on mean gain +3SD of control population)	23/74 (31%)	Regression: 42/74 (57%) Persistence: 25/74 (34%) Progression: 7/74 (9%)
Ravaioli 2017 [26]	CIN 1–3, histological diagnosis	8*	Paraffin-embedded biopsies	0 m – 10y	Colposcopy with biopsy or conization	Yes	No	Min 60 cells	> 2 signals in >10% of cells, pragmatic	3/8 (38%)	Low grade CIN: Remission: 1/3 (33%) Persistence: 1/3 (33%) Progression: 1/3 (33%) High-grade CIN: Regression: 1/5 (20%) Persistence: 3/5 (60%) Progression: 1/5 (20%)

*only those patients were included that were not treated at baseline

^a^CIN

^b^low-grade squamous intraepithelial lesion

^c^atypical squamous cells of undetermined significance

### Study Results

The main study results are summarized in Table 2. A total of 407 patients were included, of which 385 patients were diagnosed with ASCUS/LSIL/low-grade CIN and 22 patients were diagnosed with HSIL or high-grade CIN. Only five patients had a histological diagnosis of high-grade CIN. Pooling of the study results was not possible, due to a marked heterogeneity in patient populations, follow-up terms and outcome-measures. Only five out of 155 patients (3.2%) without *3q26* gain showed disease persistence or progression.

**Table 2:** Results of studies on *3q26/hTERC* gain as a prognostic biomarker in CIN

Author	Gain of *3q26/hTERC* per outcome group (using study threshold)	Progression in gain-negative group	Test properties	
			Prediction of persistence/ progression vs regression	Prediction of progression vs regression/ persistence
Heselmeyer-Haddad 2005 [19]	Only 3q26 gain: Progression: 7/12 Regression: 0/10	5/15	Sens^a^ 100% Spec^b^ 70% PPV^c^ 80% NPV^d^ 100%	
	*3q26* gain and/or tetraploidy: Progression: 12/12 Regression: 3/10	0/7	*(progression* vs *regression, only gain + tetraploidy group)*	
Alameda 2009 [20]	6 months: Regression: 7/15 Persistence/progression: 12/15	NR	6 months: Sens 80% Spec 53% PPV 63% NPV 73%	
	12/24 months Regression: 8/18 Persistence/progression: 6/8		12/24 months: Sens 75% Spec 53% PPV 43% NPV 91%	
Jalali 2010 [21]	Regression/persistence: 7/36 Progression: 10/11	1/30		Sens 91% Spec 81% PPV 59% NPV 97%
Lan 2012 [22]	Regression: 2/20 Persistence: 12/21 Progression: 13/13	0/27	Sens 74% Spec 90% PVV 93% NPV 67%	Sens 100% Spec 66% PPV 48% NPV 100%
Rodolakis 2012 [23]	Regression/persistence: 5/37 Progression: 3/3	0/32		Sens 100% Spec 89% PPV 50% NPV 100%
Obermann 2013 [24]	Regression: 16/67 Persistence: 15/55 Progression: 7/10	3/54	Sens 70% Spec 76% PPV 30% NPV 94% *(progression* vs *regression)*	Sens 35% Spec 76% PPV 58% NPV 54%
Li 2014 [25]	Regression: 4/42 Persistence/progression: 19/32	NR	Sens 59% Spec 90% PPV 82% NPV 75%	
Ravaioli 2017 [26]	Regression: 0/2 Persistence: 2/4 Progression: 1/2	1/5	NA^e^	

^a^sensitivity

^b^specificity

^c^positive predictive value

^d^negative predictive value

^e^not applicable

### Summary and Appraisal

All studies identify *3q26/hTERC* gain as a potential prognostic marker in cervical precancerous lesions. *3q26/hTERC* gain seems more frequent in persistent or progressive lesions, but positive predictive values are generally low: patients with *3q26/hTERC* gain often show disease regression during follow-up. Negative predictive values are consistently higher: absence of *3q26/hTERC* gain seems to be a strong predictor of disease regression.

Nevertheless, several limitations of the individual studies and their review must be noted. Patient populations were generally small. Most studies included only patients with low-grade lesions, which limits the evidence on the prognostic properties of *3q26/hTERC* gain in high-grade lesions. The baseline diagnosis was not determined uniformly: some studies included patients based on cytology, whereas others included only histologically confirmed lesions. Furthermore, follow-up periods and methods differed: both cytology and histology was applied. Regarding the FISH analysis, the different studies did not apply a similar signal interpretation method and threshold for gain. Another important limitation in the interpretation of the study results is that HPV testing was not performed or reported in most studies. It is therefore unclear whether all lesions were HPV-induced. This limits the applicability of the study results to high-grade lesions, which are usually HPV positive. Moreover, HPV genotype is an individual predictor in the natural history of CIN lesions. As such, information on HPV status would improve the interpretation of the study data.

Despite all limitations, *3q26/hTERC* analysis has been consistently identified as a prognostic marker in cervical precancerous lesions, with a high negative predictive value in mostly low-grade lesions. As such, evidence indicates a potential predictive role of *3q26/hTERC* gain in the natural prognosis of cervical dysplasia, but clinical applicability is yet limited and the evidence on the predictive properties of *3q26/hTERC* gain in histologically confirmed high-grade lesions is scarce (*n* = 5). This prompted us to perform a pilot study evaluating the predictive properties of *3q26/hTERC* gain in histologically confirmed high-grade lesions.

## Pilot Study: Results

### Patient Characteristics

A total of 19 women were included in our study. The mean age was 31 years (range 25–41). The mean interval between the initial colposcopy and the follow-up colposcopy with LEEP was 115 days (91–154 days). Nine women (47%) showed disease regression during this follow-up period and ten did not (53%). The mean age and biopsy-LEEP interval did not differ significantly between women who showed spontaneous regression and those who did not (mean age 32 vs 31 years, mean biopsy-LEEP interval 115 vs 114 days respectively).

### Lesions Characteristics: CIN Grade, Lesions Size, HPV Genotyping and p16 Staining

Sixteen patients were diagnosed with a CIN3 lesion and three with a CIN2 lesion. Of the patients with a CIN2 lesion, two showed regression and one showed disease persistence (22 vs 10%, *p* = 0.5). In the original study, lesions were classified according to size in two categories: larger than 2.5 mm or equal to or smaller than 2.5 mm. There was no difference between these two categories in terms of the number of women with and without regression: a lesion larger than 2.5 mm was found in 5/9 (56%) women with disease regression and in 7/10 (70%) women with disease persistence (*p* = 0.54). All women carried high-risk HPV. HPV-16 was found in 5/9 (56%) women with disease regression and 7/10 (70%) women with disease persistence (p = 0.54). The biopsy material of all cases was p16 positive, confirming HPV infection.

### FISH Results

**Fig. 1:**
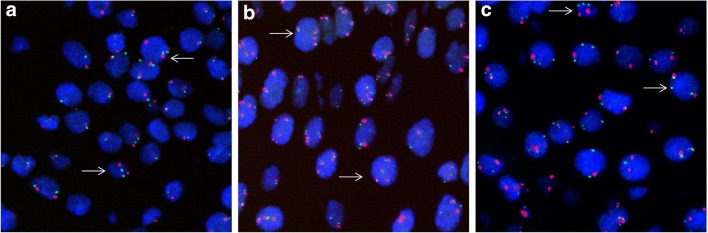
Fluorescence in situ hybridization in high-grade CIN. *Typical examples showing a disomy in *(***a)****for both 3q26 (green FITC signal) and 3C (red TRITC signal) (case number 197). In *(***b****) a tetrasomy (case 192) and in (****c****) an imbalance between 3q26 and 3c (case 218). In the latter case the cells showed a ratio of 3 to 2 signals for 3q26 and 3c respectively, with nuclei with multiple copies for both targets (classified as gain). The arrows point to the nuclei with the typical signal distribution for the cases with no gain (****a****) and gain (****b c****)*

Results of the *3q26* analysis are shown in Table 3. Figure 1 shows typical examples of the FISH analysis. Four patients showed no *3q26* gain, all of their lesions regressed spontaneously. Of interest, all CIN2 lesions showed *3q26* gain. The test performance of *3q26* gain in the prediction of natural prognosis of high-grade CIN in the study population is shown in Table 4. When the analysis was restricted to only CIN3 lesions, the positive predictive value increased to 75%, while the negative predictive value remained 100%.

**Table 3:** Results of *3q26* analysis and natural prognosis in 19 patients with high-grade CIN

Case	*3q26* copy numbers	3c copy numbers	3q status	Regression (yes/no)
96	4	4	Gain	No
125	3, 4	3,4	Gain	No
159	4	4	Gain	No
164	4	4	Gain	No
170	2–4	2–4	Gain	No
171	2–6	2–6	Gain	No
182	3, 4	3, 4	Gain	No
218	6–8	3,4	Gain	No
221	2–4	2–4	Gain	No
225	2–4	2–4	Gain	No
187	2–4	2–4	Gain	Yes
192	4	4	Gain	Yes
207	3	3	Gain	Yes
222	2–4	2–4	Gain	Yes
237	3, 4	3, 4	Gain	Yes
194	2	2	No gain	Yes
197	2	2	No gain	Yes
200	2	2	No gain	Yes
206	2	2	No gain	Yes

**Table 4:** Test performance of *3q26* gain in the prediction of natural prognosis of high-grade CIN in 19 patients

	Persistence	Regression	*Total*
Gain of 3q	10	5	*15*
No gain of 3q	0	4	*4*
*Total*	*10*	*9*	*19*
*p* = 0.0325			
Sensitivity:		100% (95% CI 66–100%)	
Specificity:		44% (95% CI 15–77%)	
Positive predictive value:		67% (95% CI 39–87%)	
Negative predictive value:		100% (95% CI 40–100%)	

## Discussion

This is the first study to assess the prognostic value of *3q26* gain as a single genetic marker in the natural prognosis of exclusively high-grade CIN. The results show that *3q26* gain is found in both women with persistence and regression of high-grade CIN, but that none of the women without *3q26* gain show disease persistence. This results in a high negative predictive value of *3q26* for disease persistence. As such, the absence of *3q26/hTERC* gain may potentially be applied to identify those lesions with a high potential of disease regression.

The test performance of *hTERC* gain in high-grade lesions in our pilot study is comparable to the test performance of *hTERC* gain in low-grade lesions, as reviewed in the literature. Negative predictive values were consistently high, while positive predictive values were much lower. The mediocre positive predictive value of *3q26/hTERC* gain as a prognostic marker for disease persistence and/or progression may indicate that *hTERC* gain is a contributing, but not critical step in cervical oncogenesis. The development of cervical precancerous lesions and subsequent carcinoma is based on a complex interaction between virus and host, in which viral oncogenic properties and the human immune system influence the cellular processes that lead to cell transformation [8, 9]. In this process, several important molecular events have been identified, among which are viral DNA integration and upregulation of telomerase [8]. However, none of these events have been identified as critical steps or ‘point of no return’. Indeed, upregulation of telomerase is not found in all high-grade CIN lesions or cervical carcinomas [12]. As such, it is unlikely that the prediction of the natural prognosis of CIN lesions will be based on one molecular event, but rather on a combination of viral, host and genetic parameters. Therefore, combining *hTERC* testing with other predictive biomarkers may lead to a test panel with a better overall test performance.

Interestingly, 3q gain can occur based on tetrasomy, in which four copy numbers are found, or aneusomy, in which three or more than four copy numbers are found. It is unclear whether there is a clinical difference between these two forms of 3q gain, in terms of the risk of disease persistence or progression. Both tetrapoidy and aneuploidy are frequent events in CIN development. The frequency of tetraploid cells is significantly increased in CIN lesions compared to normal cervical tissue and is considered an early event in cervical carcinogenesis [27]. Aneuploidy is more often found in more advanced lesions and cervical carcinoma [15]. Although it is still debated whether aneuploidy results from genomic instability of diploid cells or from chromosomal losses from tetraploid cells, evidence in CIN lesions suggests that aneuploidy is preceded by tetraploidy [27]. This would indicate that aneuploidy in CIN lesions associates with later stages of cervical oncogenesis, possibly indicated a more high-risk CIN lesion. Based on these findings, one may argue that *3q26/hTERC* gain based on aneuploidy imposes a greater risk of disease persistence or progression than *3q26/hTERC* gain based on tetraploidy. In our study, the only purely aneusomic lesion showed disease regression. Our study shows no difference in disease regression or persistence based on *3q26* gain in tetrasomic or aneusomic lesions, but numbers are small and the follow-up term was relatively short. The reviewed studies show conflicting results regarding the prognostic value of 3q gain based on either aneuploidy or tetraploidy. One study showed a positive predictive value of 100% for gain based on aneuploidy for disease progression [19]. Two other studies could not confirm this finding, but compared progression to non-progression (including persistence), which makes comparison of the studies difficult [21, 23]. Interestingly, Lan et al. found a higher progression risk for tetraploid lesions [22]. In conclusion, current evidence shows that lesions with *3q26* gain based on both tetrasomy and aneusomy can show either regression, persistence or progression. Based on these results, it remains unclear whether there is a clinical difference between *3q26/hTERC* gain based on tetrasomy or aneusomy, in terms of the risk of disease persistence or progression.

Only five out of eight reviewed studies performed HPV typing, of which only one study reported on the association between HPV and 3q gain: a non-significant association was found between viral load and 3q gain [20]. As discussed before, this limits the overall interpretation of the study results, since high-risk HPV in itself is a risk factor for disease progression/persistence. Regarding the relation between HPV infection and *3q26/hTERC* gaing, it is debated whether *3q26/hTERC* gain a direct cause of HPV infection, or an independent risk factor in high-grade CIN. It has been shown that genomic integration of HPV (with increased expression of E6 and E7) and gain of *hTERC* are important associated genetic events in the progression of CIN to cervical cancer [16]. On the other hand, 3q gains have also been detected in non-HPV-associated squamous cell cancers of the lower genital tract and other malignancies [12]. Assessment of HPV status is therefore vital in future studies on the prognostic properties of *3q26/hTERC*. Furthermore, future studies should clarify the association between HPV genotype and 3q gain, with regard to the natural prognosis of high-grade CIN.

Limitations of the current clinical study include the small patient population. The patient population was extracted from a historical cohort of patients from a previous study, based upon the availability of sufficient biopsy material. Another limitation of our study may be the use of histological specimen instead of cytology for the FISH analysis, which has been shown to be more sensitive to the identification of cells with 3q gain [12, 19]. We however chose to perform FISH analysis in biopsy material, as histology is the golden standard for a CIN diagnosis. A limitation regarding the interpretation of the study results, is the effect of a diagnostic biopsy on the natural history of the lesion. It is suggested that the biopsy itself may induce lesions regression. This would limit the interpretation of any prognostic marker, and applies to all studies on the natural history of CIN lesions. On the other hand, high-grade lesions are clinically diagnosed with a biopsy, making the prognostic effect of a prognostic biomarker clinically applicable despite the effect of the biopsy on regression itself. Another general limitation with regard to the interpretation and application of histological biomarkers in high-grade CIN is the possibility of false negative results due to sampling error, in which the biopsy is not representative of the actual disease status. We therefore propose that histological biomarkers should be applied as part of a biomarker profile, which should also contain biomarker that are independent of the disease histology. Examples are cytological, serological or epidemiological markers, such as HPV-genotype, immune markers and smoking status.

In conclusion, the results of the current review and pilot study show that the absence of *3q26* gain could potentially serve as a prognostic biomarker for the identification of CIN lesions with a high probability of disease regression, preferentially as part of a broader biomarker profile. As such, *3q26* staining could aid in the selection of women with low-grade lesions who would not need immediate colposcopic assessment and women with high-grade lesions who would not need immediate treatment. Both strategies could result in reduced costs, patient burden and side effects of surgical treatment. To confirm this hypothesis further research is necessary. Research should focus on identification of a generalized methodology for *3q26* gain testing and interpretation. Subsequently, its prognostic properties should be confirmed in a larger patient population. Moreover, assessment of the association between HPV and *3q26* gain is needed. Upon confirmation of its prognostic properties, *3q26* staining could be considered as part of a biomarker profile to triage women with high-grade lesions for conservative follow-up measures.

## Abbreviations

CIN: Cervical Intraepithelial Neoplasia
PPV: Positive predictive value
NPV: Negative predictive value
HPV: Human papillomavirus
*hTERC*: Human telomerase RNA gene
LEEP: Loop electrosurgical excision procedure
FISH: Fluorescence in situ hybridization
ASCUS: Atypical squamous cells of undetermined significance
LSIL: Low grade squamous intraepithelial lesion
HSIL: High grade squamous intraepithelial lesion
